# Retraction: Effects of pneumatic compression therapy on wound healing in patients with venous ulcers: A meta‐analysis

**DOI:** 10.1111/iwj.70022

**Published:** 2024-08-09

## Abstract

Xu Q, Li Z. Effects of pneumatic compression therapy on wound healing in patients with venous ulcers: a meta‐analysis. *Int Wound J*. 2023; 21(3): e14438. 10.1111/iwj.14438

The above article, published online on 07 November 2023, in Wiley Online Library (http://onlinelibrary.wiley.com/), has been retracted by agreement between the journal Editor in Chief, Professor Keith Harding; and John Wiley & Sons, Ltd. It came to the publisher's attention from a third party that a number of articles shared concerning similarities in format and structure. Following an investigation by the publisher, the retraction of this article has been agreed on because the peer review and publishing process for this article were found to have been manipulated. The authors did not respond to our notice of retraction.

Xu Q, Li Z. Effects of pneumatic compression therapy on wound healing in patients with venous ulcers: a meta‐analysis. *Int Wound J*. 2023; 21(3): e14438. 10.1111/iwj.14438

The above article, published online on 07 November 2023, in Wiley Online Library (http://onlinelibrary.wiley.com/), has been retracted by agreement between the journal Editor in Chief, Professor Keith Harding; and John Wiley & Sons, Ltd. It came to the publisher's attention from a third party that a number of articles shared concerning similarities in format and structure. Following an investigation by the publisher, the retraction of this article has been agreed on because the peer review and publishing process for this article were found to have been manipulated. The authors did not respond to our notice of retraction.

